# Electrographic monitoring for seizure detection in the neonatal unit: current status and future direction

**DOI:** 10.1038/s41390-024-03207-2

**Published:** 2024-04-29

**Authors:** Mary Anne J. Ryan, Atul Malhotra

**Affiliations:** 1 INFANT Research Centre, University College Cork, Cork, Ireland; 2 Department of Paediatrics and Child Health, University College Cork, Cork, Ireland; 3 Monash Newborn, Monash Children’s Hospital, Melbourne, Australia; 4 Department of Paediatrics, Monash University, Melbourne, Australia

## Abstract

**Abstract:**

Neonatal neurocritical intensive care is dedicated to safeguarding the newborn brain by prioritising clinical practices that promote early identification, diagnosis and treatment of brain injuries. The most common newborn neurological emergency is neonatal seizures, which may also be the initial clinical indication of neurological disease. A high seizure burden in the newborn period independently contributes to increased mortality and morbidity. The majority of seizures in newborns are subclinical (without clinical presentation), and hence identification may be difficult. Neuromonitoring techniques most frequently used to monitor brain wave activity include conventional electroencephalography (cEEG) or amplitude-integrated EEG (aEEG). cEEG with video is the gold standard for diagnosing and treating seizures. Many neonatal units do not have access to cEEG, and frequently those that do, have little access to real-time interpretation of monitoring.

**Impact:**

- EEG monitoring is of no benefit to an infant without expert interpretation.
- Whilst EEG is a reliable cot-side tool and of diagnostic and prognostic use, both conventional EEG and amplitude-integrated EEG have strengths and limitations, including sensitivity to seizure activity and ease of interpretation.
- Automated seizure detection requires a sensitive and specific algorithm that can interpret EEG in real-time and identify seizures, including their intensity and duration.

## Introduction

Neonatal neurocritical care is dedicated to safeguarding the newborn brain and prioritises clinical practices that promote early identification, diagnosis and treatment of brain injuries and maximise normal neurodevelopmental outcomes. Neonates are most susceptible to seizure development due to brain immaturity and a high risk of injury. The incidence of neonatal seizures in term-born infants is 1–5.5 per 1000 live births in high-income countries and as high as 39.5 per 1000 live births in low-income countries.^1^ Frequently, seizures are the first and sometimes the only clinical sign of neurological disease in neonates^2^ occurring as a result of a disruption to the regular equilibrium between excitatory and inhibitory neurotransmission in the brain.^3^

An electroencephalogram (EEG) measures the electrical activity of the brain. EEG measures the differences in electrical voltage and plays a key role in seizure detection and in monitoring the efficacy of treatment.^4^ American Clinical Neurophysiology Society (ASNS) identifies continuous conventional electroencephalography (cEEG) with video as the gold standard of care for the diagnosis and management of neonatal seizures and neurological conditions.^5^ In neonatology, EEG is an especially useful safe and unobtrusive cot-side neuromonitoring tool in the NICU (with minimal burden to the infant) providing continuous real-time monitoring of the electrical activity of the brain with video-supporting behaviour observation of the critically ill infant.^6^ EEG also supports the observation of ongoing cerebral responses to asphyxia and determines the severity of neonatal encephalopathy and the efficacy of treatment.^7^

This manuscript provides a broad overview of cEEG and amplitude-integrated EEG (aEEG) based neuromonitoring and its role in seizure detection in the neonatal unit. We will discuss seizure aetiology and the impact of seizure burden on developmental outcomes. Electrographic monitoring and seizure presentation on cEEG and aEEG will be described and incorporate the strengths and limitations of each mode of EEG monitoring (Table 1). Finally, we will describe seizure treatment before looking to the future of EEG monitoring and interpretation.

**Table 1:** Strengths and limitations of cEEG and aEEG

cEEG	aEEG
**Strengths**	
Safe non-invasive cot-side monitoring tool - minimal burden to the infant	Safe non-invasive cot-side monitoring tool- minimal burden to the infant
Displays second-by-second brain wave activity	A simplified tool that provides a visual trend over time
Standardised multi-channel electrode placement	Easy to apply and interpret with minimal training of neonatal staff
Identifies the exact timing of seizure onset	Standardised electrode placement (with limited options)
Real-time observation of response to treatment	A simplified tool providing a trend of activity over time
Provides additional information on sleep organisation, degree of maturity and abnormal brain wave features if present	Artefacts including handling may cause an upward deflection of aEEG recording and be misinterpreted as a seizure
Sensitive to localised and low-amplitude seizures of short duration that may not propagate	Easily interpreted with adequate training and complements cEEG
May reduce the use of antiseizure medication	Identify infants who warrant cEEG monitoring and therapeutic hypothermia
**Limitations**	
Requires access/maintenance of expensive equipment	Requires access/maintenance of expensive equipment(but less so than cEEG)
Requires access to expert neurophysiologist for interpretation	May miss seizures of short duration and low amplitude localised seizures
Specialist EEG technicians/ trained personnel are required to apply electrodes	Timing and duration of seizure are vaguer and poorly determine brain maturity
Ongoing costs in terms of supplies to provide the service	Real-time observation of response to treatment is vague in comparison to cEEG
Increased number of electrodes greater risk of impacting skin integrity	Limited electrodes and sensitivity only to the area beneath electrodes
Access to cEEG is the exception rather than the rule	Information on brain wave activity limited to amplitude

## Seizure aetiology

The aetiology of seizures is an important determinant of outcome and may be classified as due to cerebrovascular, metabolic, infection, developmental or genetic causes. Seizures in the term-born infant predominantly reflect an acquired cerebrovascular injury, the main cause being hypoxic-ischaemic encephalopathy (HIE, 38%), stroke (18%) or intracranial haemorrhage (12%).^8,9^ HIE is the most common cause of death and long-term disability in neonates. In a study conducted by Glass et al.^8^ seizures due to epilepsy occurred in 6% of infants with epileptic encephalopathy, 3% due to benign familial neonatal epilepsy and 4% due to brain malformation. Other causes of seizures identified include metabolic disorders (3%) and infection (4%).

Genetic epilepsies are associated with pathogenic variants with a definitive diagnosis requiring investigations including the integration of molecular biology (gene panels, chromosomal microarray and targeted gene testing), bioinformatics and clinical knowledge. Developmental causes related to cortical malformations or inborn errors of metabolism require long-term treatment and ongoing surveillance.^8,10,11^

Maternal medical, obstetric and perinatal history and placental examination may contribute to identifying the cause of seizures. Physical examination of a newborn may identify features including dysmorphism, which may lead to consideration of chromosomal abnormality, The size and quality of the fontanelle and or the presence of macrocephaly may give rise to a suspicion of defective structural brain development, interventricular haemorrhage or meningitis and will guide further investigation, management of the seizure activity^12^ and provide information on responsiveness to treatment.^12–14^

## Seizures burden and developmental outcome

Infants with moderate to severe HIE (*n* = 47) at 24–48 have over a nine-fold risk of abnormal outcome (odds ratio [OR] 9.56; 95% confidence interval [95% CI] 2.43–37.67) if the neonate has had a total seizure burden of more than 40 minutes (*p* = 0.001).^15^ Basti et al. supported these findings amongst a group of infants with moderate to severe HIE (*n* = 30) describing a statistically significant correlation between high seizure burden (*p* = 0.0004) and poor outcome.^16^ Glass et al.’s study evaluated 143 infants with HIE one-third of whom had clinically identified seizures.^17^ Glass et al. concluded the degree of neurological sequelae at 4 years increased with an increase in the severity of seizures. Uria-Avellana et al. reported that adverse sequelae from seizures are ~46% (range: 27–55%) and proposed that the strongest predictor of outcome for infants with seizures is the underlying cause and the EEG activity.^18^ Alharbi et al.’s study described higher seizure burden amongst infants with neonatal encephalopathy to be independently associated with lower cognitive (−0.21, 95% CI −0.33 to −0.08, *p* = 0.002) and language (−0.25, 95% CI −0.39 to −0.11, *p* = 0.001) at 18 months.^19^

A continued abnormal EEG, abnormal MRI and a high seizure burden is frequently linked to increased mortality and to negative neurodevelopmental outcomes.^16,20,21^ Hence electrographic monitoring is of prognostic value in determining long-term developmental outcomes.^22–26^ Application and interpretation of EEG may assist in distinguishing epilepsy from acute symptomatic seizures with implications for targeted treatment.^11^

## Electrographic monitoring

All EEG monitoring share a common set of core components, including electrodes (which may be needle electrodes, gel electrodes or cup electrodes), one amplifier and a computer interface displaying EEG recording. Rubbing the skin with a cotton tip stick and Nu-prep gel prepares the skin for electrode application. This removes residue or dead cells on the scalp, ensuring good electrode contact. A water-soluble conductive fixative paste (Ten20TM, Covidien) may be used with the primary purpose of conducting an electrical signal. A hat may be used, which is aimed at reducing electrode displacement.

### Electrode placement

**Fig. 1:**
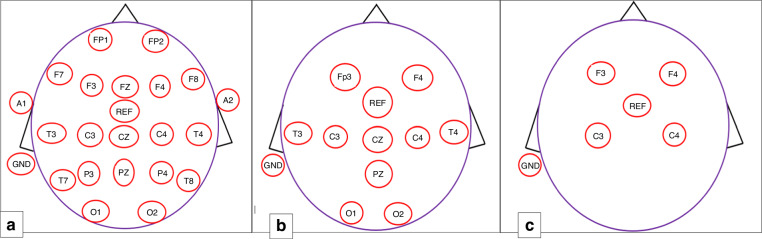
Montages that may be used when recording EEG and aEEG. **a** This montage may be used in neonates and young children. **b** A reduced neonatal montage is proportionate to term neonatal skull size. **c** Electrode placement for aEEG using minimal electrodes.

To ensure standardisation of EEG electrode placement, recordings adhere to an international electrode placement system referred to as the 10/20 system^27^ (examples see Fig. 1). This system serves as a reference map for electrode placement (montage) with the distance between electrodes proportionate to skull size and shape and covering all underlying brain regions.^5^ In newborn infants, a full 10–20 montage (≥16 electrodes) leaves minimal space between electrodes. The ACNS has identified a reduced neonatal montage (9 electrodes) as sufficient for the assessment of background EEG activity in the newborn^14^ (Fig. 1b). This version is used until the baby is full term or at most 46 weeks postmenstrual age.

Tekgul et al. carried out a study comparing the identification and characterisation of seizures based on a reduced neonatal montage (9 electrodes) versus a full montage (16 electrodes).^28^ (Fig. 1a) The number of seizures and their duration were identified by two independent reviewers with a high inter-rater agreement of 94.7% (kappa 0.85, *P* < 0.001). Tekgul concluded that the reduced montage is a sensitive method for seizure identification in comparison to a full head montage with a seizure detection sensitivity of 96.8% and 100%, respectively (seizures were, however, missed in one patient).

aEEG may be recorded from a single interhemispheric bipolar aEEG recording may include central (C3-C4) or parietal (P3-P4) electrodes (2 electrodes) or one channel on each hemisphere i.e. centroparietal electrodes (C3-P3, C4-P4) or front-central electrodes (Fp3-C3, Fp4-C4) (4 electrodes) (Fig. 1c). Variation in montage may be evident between institutions which may include number of non-cerebral channels including electrocardiograph and respiratory monitoring ± electrooculography (EOG) monitoring eye movement and electromyography (EMG) monitoring facial movements.

### Electrode impedance

Electrode impedance reflects the opposition to the flow of the electrical current. It is used as a measure of the quality of electrode contact with the skin. A low impedance (<10 kOhm’s on all electrodes) will reduce electrical noise on the recording and is recommended before commencing EEG monitoring. EEG impedance may be affected by reduced interelectrode distance, electrode placement and localised oedema at the site of electrode placement.

### EEG artefact

One of the disadvantages of electrographic monitoring lies in its sensitivity to artefact (Table 1) or undesirable signals that can interfere with the features displayed on EEG, which may be caused by physiological or environmental factors. cEEG is more sensitive to artefact than aEEG.(Table 1) Physiological sources of artefact include but are not limited to, head, eye, facial or gross body movement causing muscle artefact, respiratory movement, and ECG/pulse artefact jaw movement, sucking, hiccups head movement, sweat. Constant rhythmical, abnormal activity that does not change or evolve is suspicious of an artefact. However, such rhythmic activity may be misinterpreted as a seizure on cEEG and aEEG.^29^ Video review may assist in eliminating/ confirming the true source of activity. Education in identifying possible sources of artefact will assist in minimising/removing same, which will support accurate EEG interpretation.

Environmental sources of artefact in the NICU include electrical devices such as ventilators and intravenous pumps, cooling equipment, handling for routine care or feeding or loud noises all of which can make electrographic monitoring difficult to interpret.

### Identifying and classifying seizures on cEEG

cEEG with video is the gold standard tool that measures the number and duration of seizures, the area of origin and the pattern of migration. The International League Against Epilepsy Task Force defined electrographic seizures as events with a sudden, paroxysmal, and abnormal alteration of activity.^30^ Pressler et al.^31^ described seizures as an electrographic event with a pattern characterised by sudden, repetitive, evolving stereotyped waveforms with a beginning and end. For the most part the definitions have remained the same but without a specific duration or the amplitude specified.

The majority of infants display repetitive startle type or even jerky movements, tremors, jitteriness or physiological myoclonus which are normal to the newborn but may complicate a diagnosis or seizures.^32^ However, these movements will cease with passive flexion, are predominantly tremulous and are not associated with apnoea/desaturations tachycardia or elevated blood pressure. Seizures may be electroclinical or electrographic (subclinical). Seizures may be localised or focal, multifocal, unilateral, or bilateral, asymmetric or symmetric.^30^

Electroclinical seizures have electrographic features accompanied by clinical manifestation. The classification of seizure types is based on their primary clinical characteristics, categorising them into motor, non-motor and unclassified.^30,31,33^ Pressler et al. describe one category of motor seizures to include (but not limited to) : (a) Clonic-type seizures as more readily identified clinically presenting as jerky movements on one or both sides of the body which are regular and involve the same muscle groups.^31,33^ (b) Tonic-type generalised seizures present as an increase in muscle contraction such as extension of the infant’s limbs, posturing and clenching of fists. (c) Infants diagnosed with HIE may exhibit less coordinated movements or automatisms such as lip smacking, mouthing, eye deviations or appearance of cycling activity. Other categories of motor seizures include epileptic spasms and myoclonic non-motor seizures include autonomic and behavioural arrest.^31^

As the majority of seizures are electrographic only with minimal or no clinical manifestation,^34^ neonatologists face challenges in making a diagnosis based on clinical evaluation alone due to various possible clinical manifestations.^35,36^ Poor inter-rater agreement and accuracy in identifying seizures have previously been described.^32^ In a study carried out by Murray et al. (*n* = 526 electrographic seizures) 34% of seizures had a clinical correlate on video with only a 9% detection rate based on clinical observation by experienced clinicians.^34^ A high rate of inter-rater agreement in classifying cEEGs and terminology was described by Wusthoff et al.^37^. Three paediatric neurophysiologists independently reviewed 60 neonatal EEG’s from three centres of term infants with HIE (*n* = 180 EEG’s). Seizures were grouped by number i.e., no seizures, 1–10 seizures, 11–20 seizures or >20 seizures. Background EEG activity was standardised based on: (1) continuity(normal continuity, discontinuous or burst suppression or uninterpretable due to status epilepticus), (2) symmetry, (3) synchrony, (4) voltage 25–50 μv (peak-to-peak amplitude while awake or in active sleep), (5) borderline low (≥10 μv but <25 μv), or abnormally low (<10 μv), (6) degree of variability, (7) sharp waves, (8) periodic and rhythmic activity and an (9) an overall classification of normal or abnormal (i.e., seizures or any interictal abnormality). Based on this classification system high rate of inter-rater agreement was described by Wusthoff (kappa of 0.93, *p* < 0.001) with perfect agreement in 95% of records (57/60).^37^

Kharoshankaya et al.’s study of infants with moderate to severe HIE (*n* = 47) undergoing therapeutic hypothermia described 62% of infants having subclinical seizures detectable electrographically only.^15^ This implies that when caring for the encephalopathic infant diagnosing seizures based on clinical presentation alone is not enough. In the absence of EEG monitoring, seizure burden may be inaccurately determined^8,31,38^ and also lead to both over- and under-treatment.^39^

**Fig. 2:**
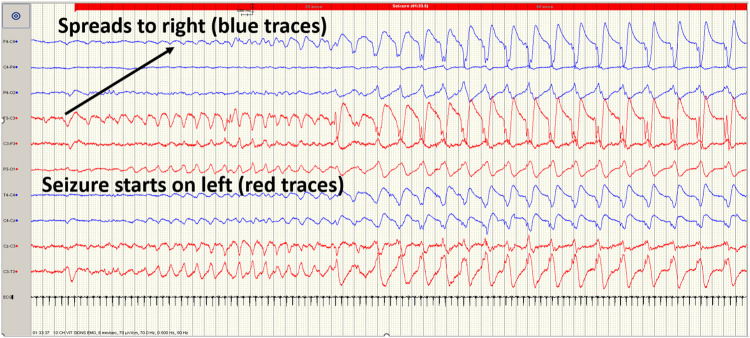
EEG of a term-born infant undergoing therapeutic hypothermia. Commencement of a seizure on the cEEG begins on the left side (red trace) with rapid frequency and low amplitude propagating to the right side (blue trace), during which amplitude increases whilst the frequency slows as the seizure progresses.

**Fig. 3:**
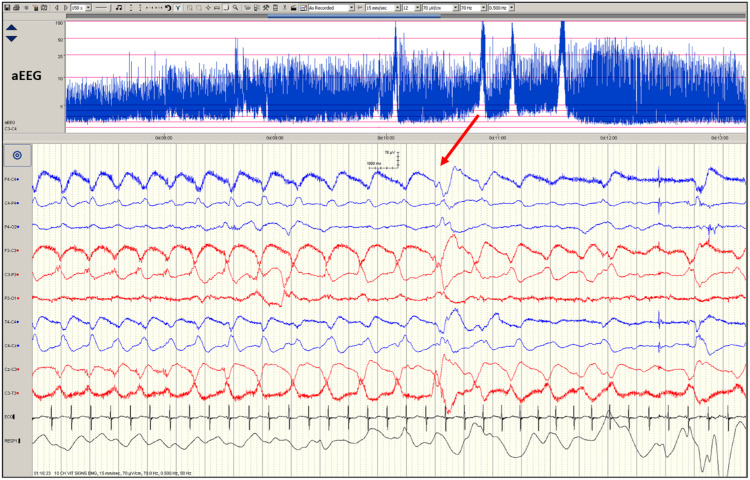
EEG shows an established seizure coming to an end, evident by a decrease in amplitude, particularly on the right side, i.e., the blue trace. The red arrow indicates the point on the aEEG when the seizure occurred, and the corresponding display of raw EEG is below.

**Fig. 4:**
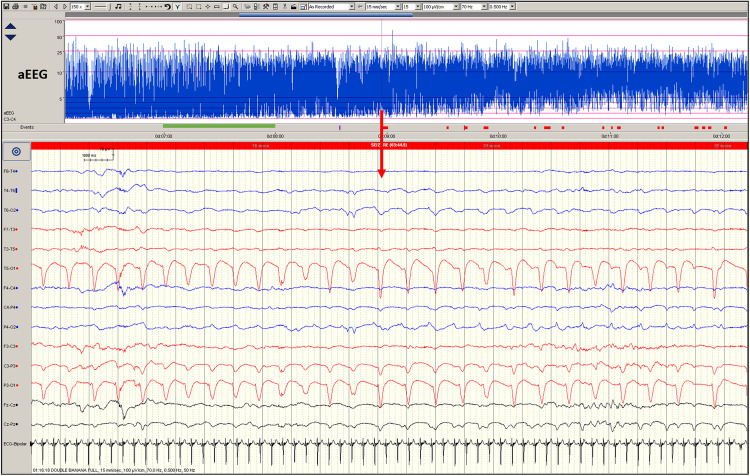
EEG of a term infant undergoing therapeutic hypothermia. Low-amplitude seizure on cEEG seen localised to the left central-occipital area. The red arrow indicates the point on aEEG where the seizure occurred and the corresponding display of raw EEG below. The seizure was not detected on aEEG (no upward deflection). The green bar on aEEG indicates one hour of EEG recording.

Seizure events must show evolution (generally increasing amplitude and decreasing frequency) and resolution of discharges over time with the discharges long enough to allow recognition of onset (Fig. 2). Evolution differentiates seizures from other rhythmic artefacts such as respiration and pulse artefact, which tend not to ‘evolve. Hence a seizure on cEEG is seen to have a beginning (Fig. 2) middle and end (Fig. 3) and can display highly diverse patterns of activity. Seizures may begin with rapid frequency and low amplitude during which the amplitude increases likely due to the progressive recruitment of neurons peripheral to the site of seizure onset whilst the frequency slows as the seizure progresses (Fig. 2). The seizure may stay localised to one area (Fig. 4) or the brain or propagate to other areas (Fig. 2). Frequency can be estimated by counting the number of peaks (or troughs) occurring in a second. The morphology may vary between and quite often within seizures on cEEG.

A high rate of inter-rater agreement in classifying cEEG’s and terminology was described by Wusthoff et al.^37^ Three paediatric neurophysiologists independently reviewed 60 neonatal EEG’s from three centres of term infants with HIE (*n* = 180 EEG’s). Seizures were grouped by number i.e., no seizures, 1–10 seizures, 11–20 seizures or >20 seizures. Background EEG activity was standardised based on: (1) continuity(normal continuity, discontinuous or burst suppression or uninterpretable due to status epilepticus), (2) symmetry, (3) synchrony, (4) voltage 25–50 μv (peak-to-peak amplitude while awake or in active sleep), (5) borderline low (≥10 μv but <25 μv), or abnormally low (<10 μv), (6) degree of variability,(7) sharp waves, (8) periodic and rhythmic activity and an (9) an overall classification of normal or abnormal (i.e., seizures or any interictal abnormality). Based on this classification system high rate of inter-rater agreement was described by Wusthoff (kappa of 0.93, *p* < 0.001) with perfect agreement in 95% of records (57/60).^37^

### Non-cerebral activity during seizures

Significant correlations have been described between cerebral and non-cerebral electrical activity.^40^ A respiratory sensor monitors respiratory rate and rhythm and is displayed on an EEG screen which may identify apnoeic episodes associated with a seizure. Electrocardiograph (ECG) monitoring displays the heart rate and rhythm on EEG screen and supports the identification of ictal tachycardia or bradycardias that may also occur during a seizure. Changes in heart rate and respirations are associated with the paroxysmal changes in the autonomic nervous system that can occur during seizures therefore, monitoring heart and respiratory variability may enhance the recognition and diagnosis of seizures.^41^ As cEEG is supplemented with synchronised video recording providing a time-locked visual display of behaviour observation of ocular and facial movements may be used (instead of EOG and EMG), which will minimise recording channels which frequently become dislodged during care and periods of fretfulness.

## Amplitude-integrated EEG

Cerebral function monitors (CFM) and EEG monitors display aEEG. aEEG is derived from conventional EEG and is a simplified compressed trend of EEG amplitude that uses a limited number of channels (Fig. 1c). The EEG signal is filtered with a bandpass of 2 Hz to 15 Hz, rectified, processed and displayed in a time-compressed semi-logarithmic scale. The semi-logarithmic scale is a linear form 0–10 μv then logarithmic from 10–100 μv and helps to attenuate the influence of high amplitude movement artefact.^42^

### Seizure presentation on aEEG

The high amplitude electrical activity during a seizure is seen as an upward deflection of the aEEG trace^43^ (Fig. 3). However, the reliability of aEEG in detecting localised seizures outside the area of neocortical tissue beneath the electrode placement, seizures of short duration (i.e., <30 seconds) and low amplitude seizures is poor due to minimal or no deflection on the aEEG (Fig. 4).^44^

### Classification and scoring systems of aEEG

Classification and scoring systems have been developed to measure background brain activity and maturation based on aEEG recording. A system based on pattern recognition was proposed by Toet et al.^45^, which includes classifications as continuous, discontinuous, burst suppression, low voltage and flat.^46^ The band displayed as the aEEG has values for upper and lower margins that represent the minimum and maximum EEG amplitude over time. In the voltage method proposed by Al Naqeeb, a normal-term and near-term aEEG trace displays a lower margin of >5 µV and an upper margin of >10 µV. A moderately abnormal aEEG displays a lower margin of <5 µV and an upper margin of >10 µV. A severely abnormal aEEG has a persistent low voltage with a lower margin of <5 µV and an upper margin <10 µV. Rennie et al. proposed that ~50% of EEG seizures may be missed on aEEG.^47^

### aEEG seizure detection sensitivity

There is a tendency to underestimate the duration of a seizure on aEEG in comparison to cEEG.^48^ Zhang et al. compared seizure detection on aEEG to seizure detection cEEG (11 channel) on a group of term infants (*n* = 62) with seizures (*n* = 876) of varying aetiology.^49^ Zhang grouped seizures by duration (510 seizures >60 seconds and 157 seizures <30 seconds on cEEG) on single-channel aEEG and identified 258 seizures longer than 60 seconds, but only 6 seizures shorter than 30 seconds. However, dual-channel aEEG identified 429 seizures lasting more than 60 seconds and 13 seizures <30 seconds.

Kadivar et al.^50^ described findings of a median sensitivity and specificity of 54% (range: 25–95) and 81% (range: 50–100), respectively on aEEG when unaccompanied by raw trace. However, the sensitivity and specificity increased to 78% (range: 68–85) and median specificity to 78% (range: 71–84) when aEEG is accompanied by raw trace. Bourez-Stewart et al. described seizures (*n* = 121) in a small group of asphyxiated infants (*n* = 12) with a detection sensitivity of 30% (C.I.: 0.22–0.38) on single-channel monitoring (C3-C4) which increased to 39% (C.I.: 0.31–0.48) when multi-channel aEEG was used.51 Falsaperia et al.’s review of the sensitivity of aEEG to seizure detection in comparison to gold standard cEEG described an overall sensitivity (single-channel, two-channel, or two-channel aEEG with raw trace EEG) ranging from 31.25% to 90%, respectively.^29^ Rakshasbhuvankar et al.^51^ described a seizure detection sensitivity of 33.7% (57/169) on aEEG which increased to 86% when ‘infants with seizures’ were included in screening for the study. However, there was a false positive rate for seizures of >50% confirmed by the use of cEEG.

Level of experience is key when interpreting aEEG.^52^ Mastrangelo et al. compared reviewing a dual-channel aEEG by neonatologists and paediatric neurologists in term newborns (*n* = 28) with seizures of varying aetiology.^53^ With a double-channel aEEG approximately half the seizures (49.4%) were identified by expert paediatric neurologists compared to 37.5% identified by neonatologists. A requirement for further and ongoing aEEG education must not be limited to neonatologists alone but must also be available to neonatal nurses who have the greatest clinical contact with the infant.

### The uItility of aEEG

**Fig. 5:**
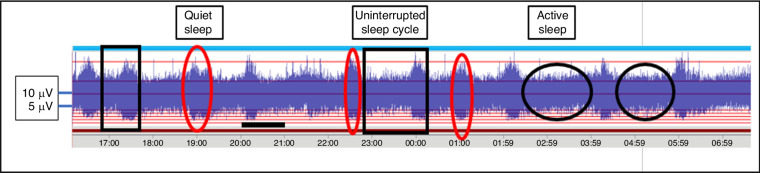
aEEG displaying sleep-wake cycling. A complete sleep-wake cycle, AS and QS are highlighted in rectangular boxes. The awake state is identified by a circle. An upper amplitude of 10 µV and a lower amplitude of 5 µV are indicated by a dashed line. Black unbroken lines indicate one hour of EEG recording.

The aEEG also provides information on cyclic variations between active sleep (AS) and quiet sleep (QS), which may be identified on aEEG from 30 to 32 weeks GA as sleep architecture emerges.^54–56^ The narrow areas of the aEEG trace represent the more consistently lower amplitude activity that occurs in AS and quiet wakefulness whilst the wider sections of the aEEG represent the alternating higher voltage bursts of activity followed by periods of quiescence or lower voltage (Fig. 5). Sleep-wake cycling is a measure of neurological wellbeing.^57,58^

The greatest clinical utility of aEEG is evident when there are limited resources and limited availability of cEEG. However, the lower seizure detection sensitivity of aEEG is not comparable to cEEG and hence aEEG is not recommended as the sole tool for seizure detection.^50^ It does however display an easily interpretable compressed visual trend of EEG and a general overview of brain wave activity which may assist in the selection of infants at risk of seizures and who may benefit from therapeutic hypothermia.^59^

Research findings also support the prognostic value of aEEG in assessing general neurological wellbeing and in determining the long-term outcome of both the term and preterm infants.^49,60^ Variane et al.^61^ suggest that all infants at risk of adverse developmental outcomes would benefit from aEEG monitoring. As interpretation is enhanced when aEEG is used in combination with dual-channel EEG,^62^ an increased number of electrodes is a better option on which to base seizure detection and management of the encephalopathic infant.

## Seizure management

Electrographic monitoring is of little benefit without the expertise to interpret the clinical significance of EEG patterns. For the most part, Infants with seizures are identified within 24 h of commencement of cEEG with video monitoring, which generally continues until 24 h seizure-free. There is a growing body of evidence suggesting seizures are damaging to neurons in human studies.^15,63^ It is unknown to what degree seizures increase damage to the brain or just reflect how a condition has developed over time. An early diagnosis of seizures may provide a window to lessen the effect of seizures on the developing brain as an early diagnosis supports better response to treatment.^64^

The timing to initiation and duration of EEG monitoring and accuracy in the identification of seizures will affect treatment.^64^ Seizure management in the newborn is focused on the use of anti-seizure medication (ASM). Jan et al.^65^ describe the higher sensitivity and specificity of cEEG with video versus aEEG hence cEEG is the preferable and gold standard neuromonitoring tool which may reduce the use of ASM. Accurate interpretation of EEG will ensure ASM is prescribed only to infants with confirmed diagnosis of seizures reducing unnecessary treatment which may cause neuronal apoptosis and potential toxicity with harmful effects^66^ and potential long-term implications for associated co-morbidities.^10,67^

Mild seizures with a decreasing presence before the administration of ASM are more likely to respond to treatment. Pavel et al. identified a significantly lower seizure burden amongst infants treated with ASM within one hour of a seizure, suggesting seizure treatment may be time-critical. This may be a major factor in reducing seizure burden, the negative effects of seizures and optimise developmental outcomes (in addition to underlying pathology).^68^ When there a suspicion of neonatal seizures based on clinical observation alone administering ASM without EEG may be standard practice in many units.^69^

ASM’s frequently lead to the uncoupling or disassociation of electroclinical seizures with persistence of electrographic seizures without clinical manifestation.^70^ Sher et al. described uncoupling occurring in up to 58% of infants (*n* = 59) after treatment with first-line medication of either phenobarbitone or phenytoin. As ASM may also be neurotoxic hence blood levels frequently monitored.^71^

Phenobarbitone is recommended as the first-line medication and effective in ~40–60% of infants. It may, however, cause both voltage depression and an increase in discontinuity. Co-morbidities and family medical history may influence choice of first-line pharmacotherapy.^69^ Choice of second-line medication is variable but may include phenytoin or carbamazepine. Intravenous benzodiazepines such as, lorazepam, clonazepam or midazolam may also be a treatment of choice.^72^ ASM’s may affect the background EEG activity^71^

## The future of seizure detection

Alone, a neonatologist’s clinical judgement of seizure activity is not enough. Developing a system that can analyse complex EEG data for seizure activity may be especially useful in institutions that do not have timely access to expert interpretation. Quantitative EEG analysis and the development of seizure detection algorithms or artificial intelligence (AI) tools remove subjectivity in interpretation.^73^ This objective mathematical computer-based approach interprets cEEG narrowing the gap between the complexity of the cEEG recording and the level of expertise required for seizure identification.^74^ AI tools are described as having a high sensitivity and specificity of >80%.^75^

**Fig. 6:**
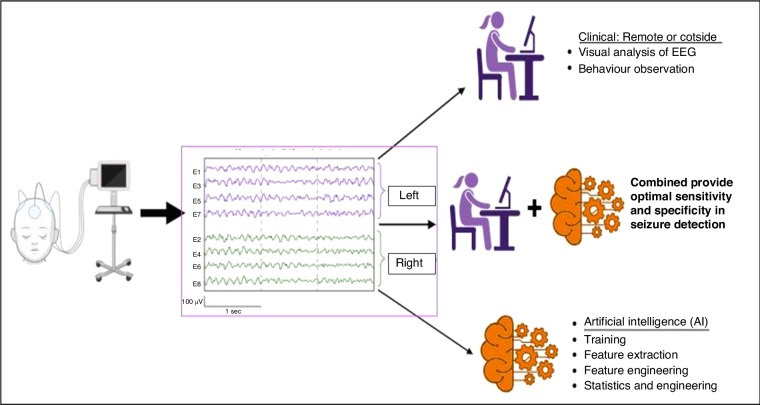
The future direction of EEG interpretation. Future interpretation of EEG will combine expert interpretation (through visual analysis and behavioural observation) with the use of artificial intelligence (AI). AI uses an objective mathematical computer-based approach to analyse EEG data thus creating intelligent machines that can interpret EEG previously dependent on human expertise alone.

Detecting seizures in the neonatal brain in a fully automated manner supports early identification and treatment of electrographic seizures. However, seizure detection algorithms are not without difficulties. Normal newborn movements and environmental artefact may be misinterpreted by the algorithm, leading to a false positive seizure detection alert. Further work needs to be done to develop intelligence that will recognise and reduce artefact-induced false positive detections.^76^ Whilst there have been significant advances in technology and artificial intelligence (AI), the combination of AI and clinical input outperforms the use of AI alone (Fig. 6).^74^

Technology requires an initial outlay but the growing field of telemedicine supports remote delivery of healthcare and the interpretation of such technology to ensure equity of access to speciality services for infants in both HIC and LMIC, Telemedicine also improves communication, and reduces dependence on trained personnel whilst lowering the costs of healthcare globally.^77^ The availability of expert cEEG interpretation 24/7 will not alone provide a more accurate measure of seizure burden but will create new opportunities to increase and enhance our service provision to infants at risk of seizures, including early identification of infants who may benefit from Therapeutic Hypothermia. Fitzgerald et al.^78^ monitored the utilisation and clinical impact of a remote cEEG monitoring service. This service was effective in the identification of seizures and improved the quality of care and treatment provided.

## Conclusion

Seizures are a neonatal emergency and detection/treatment plays a major part in neurocritical care. The aetiology of seizures and seizure burden are associated with long-term outcomes. The methods used to monitor infants at high risk of seizures such as encephalopathic infants, combine subjective and objective measures using gold standard cEEG with video, and aEEG. Despite an increased focus on neonatal neurocritical care, neuromonitoring remains challenged by the availability of technology and the resources to ensure timely access and interpretation of EEG by expert neurophysiologists.

With regards to seizure monitoring and seizure detection in the neonate, three key issues must be considered:

1. EEG monitoring is a reliable cot-side neuromonitoring tool and of diagnostic and prognostic use, but is of no benefit to the infant if timely interpretation is not available.
2. cEEG and aEEG are used variably in units across the world. Both have strengths and limitations including sensitivity to seizure activity and ease of interpretation.
3. Automated seizure detection algorithms are beneficial to units with and without access to expert interpretation.

## Data Availability

Data sharing is not applicable to this article as no datasets were generated or analysed during the current study.

## Materials availability

The material compiled in this manuscript has not been submitted elsewhere for publication.
